# Proteomics Analysis of Antitumor Activity of *Agrimonia pilosa* Ledeb. in Human Oral Squamous Cell Carcinoma Cells

**DOI:** 10.3390/cimb44080229

**Published:** 2022-07-25

**Authors:** Tae-Young Kim, Kwang-Soo Koh, Ji-Min Ju, Yeon-Ju Kwak, Soo-Kyung Bae, Hye-Ock Jang, Da-Sol Kim

**Affiliations:** 1 Department of Dental Pharmacology, School of Dentistry, Pusan National University, 49, Busandaehak-ro, Yangsan-si 50612, Korea; lotto0202@hanmail.net (T.-Y.K.); skkkwang@naver.com (K.-S.K.); wnwlals88@gmail.com (J.-M.J.); skbae@pusan.ac.kr (S.-K.B.); 2 Education and Research Team for Life Science on Dentistry, Pusan National University, 49, Busandaehak-ro, Yangsan-si 50612, Korea; 3 Research Institute of GagopaHealing Food BioFarm, Agricultural Corporation Gagopa Healing Food Co., Changwon 51219, Korea; kyjred@hanmail.net; 4 Dental and Life Science Institute, School of Dentistry, Pusan National University, 49, Busandaehak-ro, Yangsan-si 50612, Korea; 5 Department of Dermatology, Pusan National University Yangsan Hospital, Yangsan-si 50612, Korea

**Keywords:** natural drug product, *Agrimonia pilosa* Ledeb., oral squamous cell carcinoma, antitumor effect, proteomic technology, drug discovery

## Abstract

Oral cancer is a malignant neoplasm of oral cavity. It accounts for approximately 5% of all malignant tumors. Approximately 97% of all oral cancers are squamous cell carcinomas, followed by adenocarcinomas, and rarely malignant melanomas. It occurs particularly in males (twice as common in males than in females) of middle age (above 40 years). *Agrimonia pilosa* Ledeb. has traditionally been known for its effective antitumor activity and is currently used in China for cancer therapy. *A. pilosa* Ledeb. has been traditionally used for the treatment of abdominal pain, sore throat, headache, blood discharge, parasitic infections, and eczema in Korea and other Asian countries. Most studies on *A. pilosa* Ledeb. are related to the leaves and a few investigated the roots of the plant. However, detailed mechanisms of antitumor activity of *A. pilosa* Ledeb. have not been fully elucidated. Furthermore, to date, there have been no reports on the antitumor effect of *A. pilosa* Ledeb. in oral squamous cells. In this study, we used proteomic technology to observe changes in proteins related to anticancer activity of *A. pilosa* Ledeb. and identified target proteins among altered proteins to reveal the underlying mechanism of action.

## 1. Introduction

Oral cancer is the sixth most common cancer among all malignancies and recurs more frequently in developing countries. Oral cancer spreads more quickly than other cancers and often leads to early death. In addition, it causes substantial psychological impact in patients due to considerable decrease in activities and aesthetic damage, particularly because it affects the most crucial part of the human body, which is adjacent to sensitive organs. Oral cancer is a collective term for cancers occurring in the oral cavity and throat, such as periodontal, buccal mucosa, lips, tongue, salivary glands, and pharynx, and more than 90% of oral cancers are epithelial cell carcinomas [1]. Its pathogenesis is anticipated to be similar to that of epithelial cell carcinomas originating in other parts of the body, but the exact mechanism is not known. 

Oral cancer is characterized by local infiltration and cervical lymph node metastasis. Due to the relatively low rate of distant metastasis, local therapies, such as surgery and radiation therapy, have been the first-line treatment [2]. Recently, combination therapies, such as combination chemotherapy, radiation therapy, and extensive resection surgery, have been studied, and the use of chemotherapy has gradually increased. Cisplatin is the most widely used chemotherapeutic agent for the treatment of head and neck tumors, including oral cancers [3,4]. However, chemotherapeutic agents cause serious side effects such as renal toxicity; thus, their application is limited. Therefore, cancer chemopreventive agents, mostly natural products, are preferable to prevent or inhibit carcinogenesis. Flavonoids, steroids, alkaloids, tannins, and phenol compounds are the representative physiological components of plants. As with other pharmaceutical agents useful for disease prevention, pharmacoeconomic analysis of cancer chemopreventive formulations should be considered, and the composition of the formulation should improve over time [5]. Anticancer effects of natural products reported to date have been mainly limited to gastric cancer [6], liver cancer [7], colorectal cancer [8], and blood cancer [9,10].

There have been few reports on anticancer effects of plant products in human oral epithelial cell carcinoma (OSCC). Considering that the efficiency of various anticancer drugs differs depending on the cancer cell type, it is essential to identify natural product extracts with reported anticancer activity in oral epithelial cell lines. 

*Agrimonia pilosa* Ledeb. (*A. pilosa*) has been traditionally used in Japan and China as an antidiarrheal, hemostatic, and antiparasitic agent [11]. Pharmacologically, *A. pilosa* exhibited anti-inflammatory activity in *Porphyromonas gingivalis* lipopolysaccharide-induced RAW 264.7 cells [12], antitumor activity in lung cancer, breast cancer, and prostate cancer cell lines through G0 cell cycle arrest [13], antiviral activity against SARS-CoV-2 [14], antioxidant effect through DPPH and superoxide radical scavenging activity [15], antibacterial activity against methicillin-resistant *Staphylococcus aureus* [16], osteogenic differentiation promotion in MC3T3-E1 cells by targeting miR-107 [17], blood coagulation function [18], and anti-hyperglycemic activity by inhibiting inflammatory response in high-fat diet-fed rats [19]. 

*A. pilosa* is a perennial plant that has been used clinically for a wide variety of maladies, including hemorrhage, diarrhea, toxification, and parasitic infections. We demonstrated anti-inflammatory effect of the plant in RAW 264.7 cells and identified several anticancer components by MS/MS analysis. These results explain why we attempted to verify the antitumor effect of *A. pilosa*. Most studies on *A. pilosa* are related to its leaves, and only few studies have investigated the roots of *A. pilosa*. However, the detailed mechanisms of antitumor activity of *A. pilosa* have not yet been fully elucidated. To date, there have been no reports on antitumor effect of *A. pilosa* against OSCC. In this study, we employed proteomics and molecular biological approaches to examine the antitumor effect of methanol extract of the roots of *A. pilosa* and altered proteins by *A. pilosa* extract to elucidate its mechanism of action. 

## 2. Materials and Methods

### 2.1. Cell Cultures

The OSCC cell line YD-10B was purchased from the Korea Cell Line Bank (Seoul, Korea). The cells were incubated in RPMI medium supplemented with 10% fetal bovine serum (FBS; GIBCO BRL, Grand Island, NY, USA) and 100 U⁄mL penicillin-streptomycin (Invitrogen, Carlsbad, CA, USA) at 37 °C in an atmosphere containing 5% CO_2_.

### 2.2. Preparation of A. pilosa Root Extract

Dried *A. pilosa* roots were purchased from With Tech M&C (Changwon, Korea). To prepare *A. pilosa* root extract (APL), 4 L of 99.8% methanol was added to *A. pilosa* roots and shaken several times over eight days. This step was repeated four times. After obtaining the crude extract, the solution was filtered using a 185-mm filter paper and concentrated under reduced pressure in a water bath. 

Subsequently, the solution was lyophilized using a freeze dryer (Labconco, MO, USA). The APL yield was 16.34%. All fractions were powdered and stored at −20 °C. In this study, lyophilized extract was dissolved in dimethyl sulfoxide. 

### 2.3. Cell Viability Assay

To evaluate the cell viability of APL, YD-10B cells were seeded in a 96-well plate at a concentration of 5 × 10^3^ cells/well and were stabilized for one day. APL (2.5, 25, and 250 µg/mL) was then added, and dimethyl sulfoxide without extract was used as control. After the treatment, the cells were cultured for 72 h. Next, 10 μL of CCK-8 was added to 100 μL of RPMI 1640 without FBS and incubated for 1 h at 37 °C and 5% CO_2_. Subsequently, absorbance was measured at 450 nm using an enzyme-linked immunosorbent assay reader (Tecan, Mannedorf, Switzerland). 

### 2.4. Cell Apoptosis Assay

YD-10B cells were seeded in a 6-well plate at a density of 5 × 10^4^ cells/well and treated with APL. After 72 h, the supernatant and adherent cells were collected. The cells were centrifuged at 3000 rpm for 5 min, the supernatant was discarded, and the pellet was resuspended in 1× binding buffer. Subsequently, 100 μL of the sample solution was transferred to a 5-mL culture tube and incubated with 5 μL of FITC-conjugated annexin V (ThermoFisher, Waltham, MA, USA) and 5 μL of propidium iodide (PI; ThermoFisher, Waltham, MA, USA) for 15 min at room temperature in the dark. Next, 400 μL of 1× binding buffer was added to each sample tube, and the samples were analyzed using FACSCalibur Cell Analyzer (BD Biosciences, San Jose, CA, USA).

### 2.5. Two-Dimensional Gel Electrophoresis (2D-GE)

YD-10B cells were treated with APL for 72 h. To prepare samples, the cells were washed twice with 1/4 diluted phosphate-buffered saline. Protein extraction and quantification were conducted at the Yonsei Proteome Research Center [20]. 2D-GE was performed as described, and 540 μg of the protein was loaded on the gel. Aliquots of the sample buffer (7 M urea, 2 M thiourea, 4.5% CHAPS, 100 mM DTE, 40 mM Tris, pH 8.8) were placed on immobilized non-linear gradient pH 3–10 strips (Amersham Biosciences, Uppsala, Sweden). Isoelectric focusing was performed at 80,000 Vh. The second dimension was analyzed on 9–16% linear gradient polyacrylamide gels (18 cm × 20 cm × 1.5 mm) at a constant current of 40 mA for each gel for approximately 5 h. After protein fixation in 40% methanol and 5% phosphoric acid for 1 h, the gels were stained with Coomassie Brilliant Blue G-250 for 12 h. The gels were destained with water and scanned using Bio-Rad GS710 densitometer (Bio-Rad Laboratories, Inc., Richmond, CA, USA), which were converted into electronic files and analyzed using the ImageMaster Platinum 5.0 image analysis program (Amersham Biosciences, Amersham, UK).

### 2.6. Real-Time Quantitative PCR Analysis

Real-time PCR was performed using ABI 7500 Instrument (Applied Biosystems, Warrington, UK). cDNA was diluted (1:10) in Power SYBR Green PCR Master Mix, and the experiment was performed according to the manufacturer’s instructions. Standard method was used for melting curve analysis. The data were analyzed using the ABI software, and the values were determined using the _∆∆_Ct method. The primer sequences used in this study are listed in Table 1. 

### 2.7. Western Blot Analysis

Protein expression was examined by western blotting. YD-10B cells were seeded at a density of 1 × 10^6^ cells/mL on 60 mm^2^ cell culture plates pre-treated with APL. The cells were incubated at 37 °C in a 5% CO_2_ incubator for 72 h and washed with cold phosphate-buffered saline. For protein isolation, radioimmunoprecipitation assay (RIPA) buffer containing protease inhibitors and phosphatase inhibitors (NaF and Na_3_VO_4_) was used. The cells were incubated on ice for 30 min. Pierce BCA Protein Assay Kit was used for protein quantification. Equal amounts of protein samples (20–30 μg) were loaded on SDS-PAGE and transferred to a PVDF membrane after electrophoresis. Primary antibodies against cleaved caspase-3 (#9661) and cleaved PARP (#9541) were used. For quantification, Luminata Forte Western HRP Substrate (Merck Millipore, Burlington, MA, USA) was added to the membrane, and the LAS-4000 (GE Healthcare Life Sciences, Piscataway, NJ, USA) imaging system was used for data analysis.

### 2.8. LC-MS/MS for Peptide Analysis

Nano LC-MS/MS analysis was performed using Easy n-LC (Thermo Fisher, San Jose, CA, USA) and LTQ Orbitrap XL mass spectrometer (Thermo Fisher, San Jose, CA, USA) equipped with a nano-electrospray source. Samples were separated on a C18 nanopore column (150 mm × 0.1 mm, 3 μm pore size; Agilent Technologies, Inc., Santa Clara, CA, USA). Mobile phase A for LC separation comprised of 0.1% formic acid and 3% acetonitrile in deionized water. Mobile phase B comprised of 0.1% formic acid in acetonitrile. The chromatographic gradient was designed to increase linearly from 0% B to 60% B in 9 min, 60% B to 90% B in 1 min, and 3% B in 5 min. The flow rate was maintained at 1800 nL/min. Mass spectra were acquired using data-dependent acquisition with a full mass scan (380–1700 *m*/*z*), followed by 10 MS/MS scans. MS1 full scans were acquired at a resolution of 15,000 in the Orbitrap using automatic gain control (AGC) target of 2 × 10^5^. For MS/MS in the LTQ configuration, AGC was set at a target of 1 × 10^4^. 

### 2.9. Database Searching

The Mascot algorithm (Matrix Science Inc., Boston, MA, USA) was used to identify peptide sequences present in a protein sequence database. Database search criteria were as follows: taxonomy, Homo sapiens (downloaded 10 April 2018); fixed modification, carbamidomethylated at cysteine residues; variable modification, oxidized at methionine residues; maximum allowed missed cleavage, 2; MS tolerance, 10 ppm; MS/MS tolerance, 0.8 Da. The peptides were filtered using a significance threshold of *p* < 0.05.

### 2.10. Heterogeneous Nuclear Ribonucleoprotein (hnRNP) siRNA Treatment

To suppress hnRNP protein expression, hnRNP siRNA was transfected using Lipofectamine 2000 reagent (Invitrogen Life Technologies, Carlsbad, CA, USA) according to the manufacturer’s instructions. When the oral cancer cell line YD-10B achieved 70–80% confluence in a 60-mm plate, the cells were treated with Lipofectamine and hnRNP siRNA (Table 2) in serum-free Opti-MEM and cultured for 5 h. Subsequently, RPMI culture medium containing 10% FBS was added, and the cells were incubated for 72 h at 37 °C and 5% CO_2_.

### 2.11. Statistical Analysis

The data in this study are representative of three independent experiments and expressed as the mean ± SEM. The values are indicated using the Wilcoxon matched-pair signed-rank test. Statistical significance was set at * *p* < 0.05, ** *p* < 0.02, and *** *p* < 0.01.

## 3. Results

### 3.1. Effects of APL Treatment on YD-10B Cell Proliferation and Apoptosis

**Figure 1:**
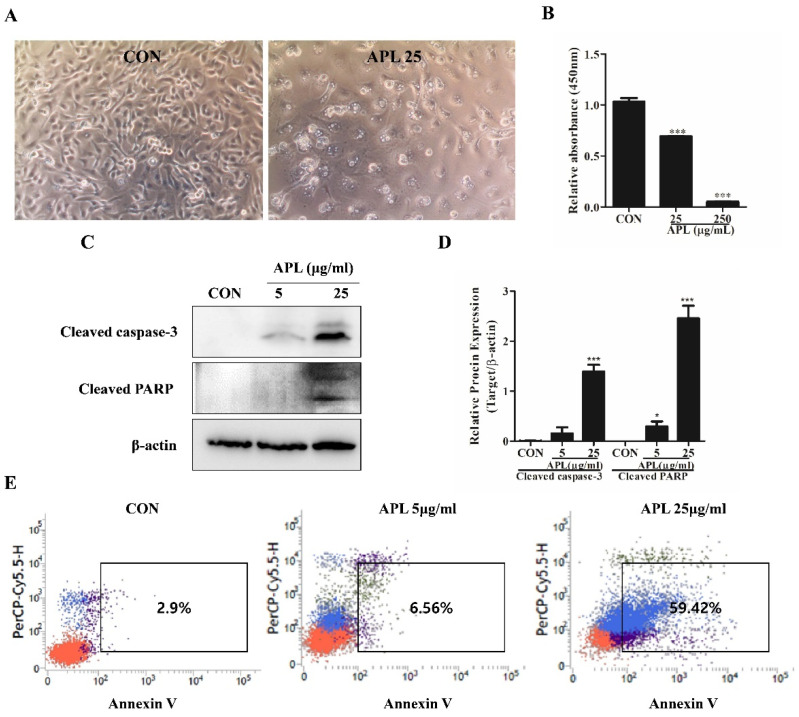
Induction of apoptosis in YD-10B cells by APL treatment. (**A**) Bright field microscope images of YD-10B cells treated with APL (25 μg/mL) and without treatment (200× original magnification). (**B**) CCK-8 assay of YD-10B cells treated with APL (25 and 250 μg/mL). (**C**) Cleaved caspase-3 and cleaved PARP expression analyzed by western blotting. (**D**) Quantification of cleaved caspase-3 and cleaved PARP expression. (**E**) Flow cytometry analysis using Annexin V/propidium iodide. Values are presented as the mean ± SEM (n = 3). * *p* < 0.05, *** *p* < 0.01.

To investigate whether APL affects the proliferation of YD-10B cells, the cells were treated with different concentrations (25 and 250 μg/mL) of APL for 72 h, and cell viability was assessed using the MTT assay. Before the CCK-8 assay, the cell morphology was observed using a phase-contrast microscope; the images revealed that APL treatment led to changes in cell morphology. As shown in Figure 1A,B, APL caused a significant dose-dependent decrease in the proliferation of YD-10B cells. At doses of 25 and 250 μg/mL, the proliferation of YD-10B cells was reduced by approximately 50% and 90%, respectively. Based on these results, 25 μg/mL of APL was used to examine its effects on YD-10B cell apoptosis; the cellular apoptotic proteins cleaved caspase-3 and cleaved PARP were analyzed by western blotting. The results indicated increased levels of these protein (Figure 1C), suggesting that APL affects cell proliferation and apoptosis at 25 μg/mL. Annexin V expression was detected using flow cytometry analysis (FACS); annexin V expression increased with increase in APL concentration in a dose-dependent manner (Figure 1D), further providing clear evidence of cell apoptosis.

### 3.2. Identification of Differentially Expressed Proteins in APL-Treated and Non-Treated YD-10B Cells

**Figure 2:**
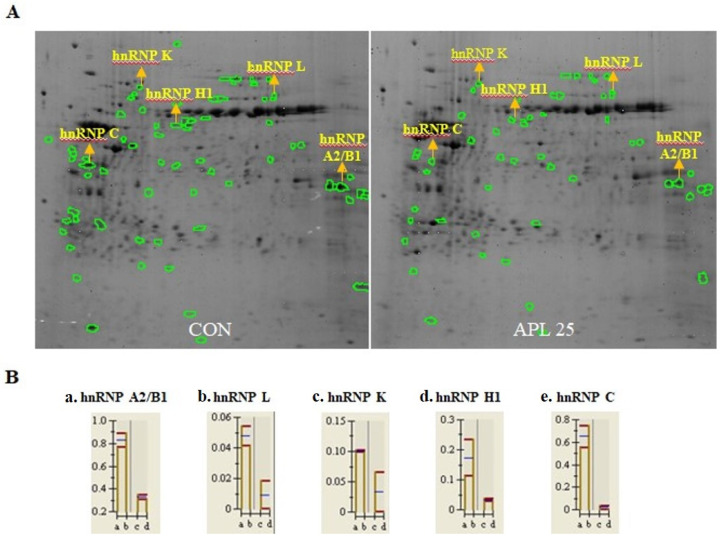
Analysis of proteins expression in YD-10B cells. (**A**). Two-dimensional gel electrophoresis analysis of proteins extracted from 25 µg/mL APL-treated and control YD-10B cells. (**B**). Quantification of two-dimensional gel electrophoresis. Analysis was performed on three independent biological replicates of proteins from both control and treated cells.

The apoptotic effect of APL was confirmed using various methods, and the results indicated that APL induced apoptosis in YD-10B cells. 2D-GE was performed to confirm the expression of proteins that regulate apoptosis. Several spots that increased or decreased significantly were observed. Approximately 167 spots per gel were detected within the pH range of 10. Thirteen spots were upregulated by more than 2-fold, whereas 25 spots were downregulated by more than 2-fold. LC/MS analysis was performed on a few selected spots (Figure 2). 

### 3.3. Effect of APL Treatment on hnRNP Family Proteins Expression in YD-10B Cells

**Figure 3:**
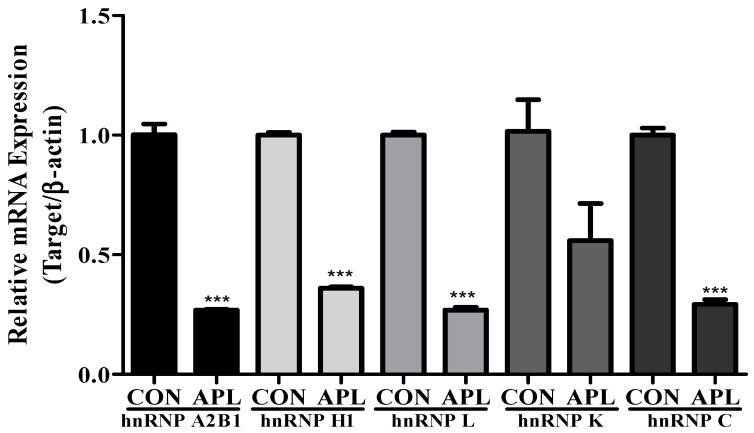
APL treatment reduced hnRNP family genes expression in YD-10B cells. YD-10B cells were treated with APL (25 µg/mL) for 72 h. Gene expressions of hnRNP A2B1, hnRNP H1, hnRNP L, hnRNP K, and hnRNP C were analyzed by real-time PCR. All values are presented as the mean ± SEM (n = 3). *** *p* < 0.01.

LC-MS/MS analysis showed decreased expression of hnRNP family proteins, including hnRNP A2B1, hnRNP L, hnRNP H1, hnRNP K, and hnRNP C. To further confirm these findings, the cells were treated with 25 μg/mL APL, harvested after 72 h, and real-time PCR was performed using the corresponding primers. The results of real-time PCR were consistent with 2D-GE results (Figure 3).

**Figure 4:**
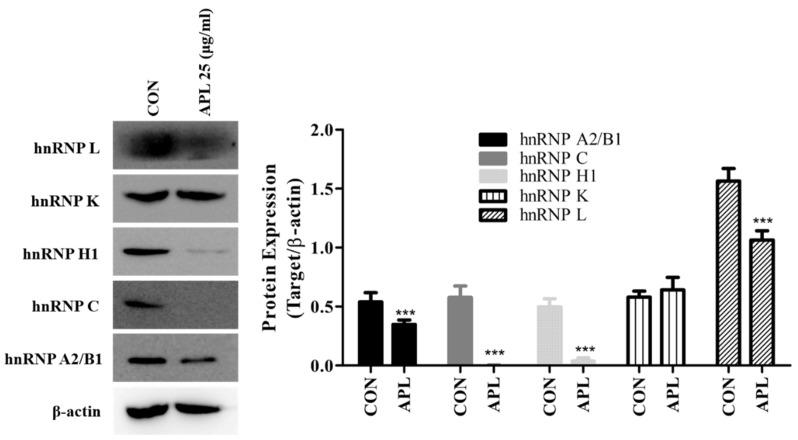
APL treatment reduced hnRNP family protein expression in YD-10B cells. YD-10B cells were treated with APL (25 µg/mL) for 72 h. Expression of hnRNP A2B1, hnRNP H1, hnRNP L, hnRNP K, and hnRNP C were analyzed by western blotting. All values are presented as the mean ± SEM (n = 3). *** *p* < 0.01.

As described earlier, the expression levels of hnRNP family proteins were determined in YD-10B cells treated with 25 μg/mL APL. To verify these results, the cells were treated with APL for 72 h and harvested to isolate the proteins. The 2D-GE results were confirmed by western blot analysis using primary antibodies against these proteins. The results of western blot analysis matched with those of 2D-GE for all hnRNP proteins except hnRNP K (Figure 4). 

### 3.4. Apoptosis Effect of hnRNP Protein Silencing in YD-10B Cells

**Figure 5:**
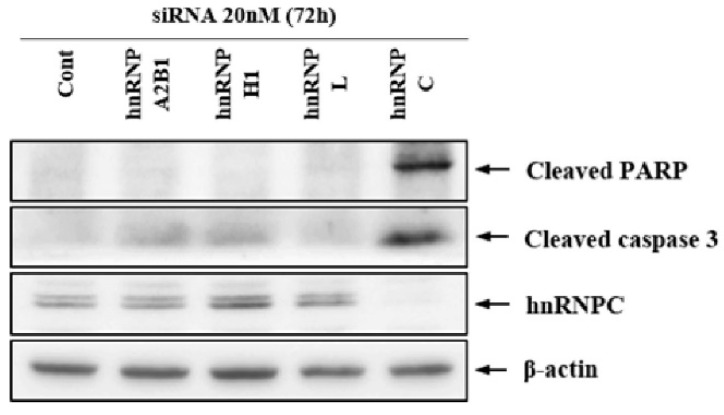
Apoptosis effect of hnRNP family protein silencing in YD-10B cells. The cells were transfected with siRNAs and incubated for 72 h. Next, siRNA efficiency was evaluated, and hnRNP C protein expression was specifically silenced using hnRNP C siRNA. Only hnRNP C siRNA-transfected group showed increased expression of the apoptosis markers cleaved caspase-3 and cleaved PARP.

To investigate the role of the hnRNP family proteins, whose expression was reduced by APL treatment, YD-10B cells were transfected with hnRNP siRNA. Several siRNA candidates were designed to conduct loss-of-function studies. Among these candidates, the most effective siRNA sequences were selected, which are listed in Table 2. siRNA-transfected cells were incubated for 72 h, and hnRNP protein expression in these cells were analyzed by western blotting. First, the selective expression of siRNA was confirmed; each siRNA-transfected sample was treated with hnRNP C, and the results indicated that only hnRNP C siRNA silenced hnRNP C expression (Figure 5). Subsequently, we observed increased expression of cleaved caspase-3 and cleaved PARP only in hnRNP C siRNA-treated group in an attempt to verify apoptosis in these samples. Based on these results, hnRNP C was identified as a potential anticancer target.

## 4. Discussion

*A. pilosa* has traditionally been known for its antioxidant, anti-inflammatory, and anti-hyperglycemic effects. The anticancer property of *A. pilosa* has been described in a few studies. Although several studies have examined and isolated chemical constituents from this plant, little evidence exists supporting the antitumor activity of individual molecules identified in these studies [13,21,22,23]. Another study reported that *A. pilosa* methanol extract inhibited the invasion of cancer cells through inactivation of ERK and JNK in HT1080 cells [21]. 

The pharmacological activity of *A. pilosa* may be attributed primarily to its phenolic compounds agrimoniin, catechin, quercetin, and rutin. *A. pilosa* has been commonly used in Korea and other Asian countries as a natural flavoring agent and to prevent or treat various diseases, such as hemorrhage, chronic fatigue syndromes, and liver disorders. Hnit et al. [13] reported *A. pilosa* as the most effective herb and further identified agrimol B as a novel compound that can impede cell cycle progression in prostate and lung cancer cells through G0 phase arrest. Miyamoto et al. [24] demonstrated that agrimoniin, a tannin present in large amounts in this plant, is the main antitumor agent. According to MS/MS analysis, (2R,3R)-taxifolin-3′-O-β-D-glucopyranside, (24S)-5α,8α-epidioxy-ergost-6-en-3β-ol, 3-(4′-hydroxy-benzyl)-5,7-dihydroxy-6,8-dimethyl-chroman-4-one, and €-hexadecyl-ferulate possessing antitumor activity have been identified in APL [12].

The MTT assay results showed that neither the viability of YD-10B cells was affected by using serum-free medium, nor it was significantly different when the cells were exposed to *APL*. Other studies have also demonstrated dose-time dependence of the inhibitory effect of *A. pilosa* on tumor cells.

In this study, proteomic techniques were used to analyze the expression of proteins released by YD-10B cells in response to APL treatment. Among 2000 protein spots detected in the 2D-GE profile of YD-10B cells grown in the absence or presence of APL, 569 proteins were paired in groups. Among the 569 pairs, 42 spots showed a 2-fold increase, and 70 spots showed a 2-fold decrease in APL-treated cells compared with that in non-treated cells.

In the group of 38 spots with more than 2-fold increase or decrease, 13 spots showed an increase, and 25 spots showed a decrease, with large differences between the increasing and decreasing spots selected for mass spectrometry. Several spots that matched peptides included heterogeneous nuclear ribonucleoprotein H3, heterogeneous nuclear ribonucleoprotein A2/B1, human translation initiation factor 3, and ras-like protein. The real-time PCR and proteomic experiments examining increased or decreased expression of proteins confirmed an increased expression of cytokeratin and a decreased expression of heterogeneous nuclear ribonucleoprotein and keratin. 

hnRNPs control maturation of newly formed heterogeneous nuclear RNAs (hnRNAs/pre-mRNAs) into messenger RNAs (mRNAs). hnRNPs act as key proteins in the cellular nucleic acid metabolism to regulate gene expression [25]. hnRNPs are RNA-binding proteins that form complexes with heterogeneous nuclear RNA (hnRNA). These proteins are associated with pre-mRNAs in the nucleus and influence processing of pre-mRNA and other aspects of mRNA metabolism and transport. While nearly all hnRNPs are present in the nucleus, some reportedly shuttle between the nucleus and the cytoplasm. LC/MS-MS analysis detected five subfamilies of hnRNPs: hnRNP A2/B1, hnRNP C (C1/C2), hnRNP H, hnRNP K, and hnRNP L. According to the study results, anticancer activity was regulated by hnRNP C induced by APL in oral cancer. In 2020, we reported an MS/MS analysis study of *A. pilosa*, and the results indicated the presence of several anticancer components. This is the first report suggesting that the anticancer effect of APL is mediated, in part, through impaired functions of the hnRNP family, based on insights obtained using a chemical proteomics strategy. 

Generally, increased mRNA levels lead to increased protein levels. However, if the increase in protein levels does not correspond to increased mRNA expression, it cannot be assumed that the drug promotes the expression of a specific gene. Therefore, real-time assessment is the most reliable method to confirm gene expression patterns. We performed western blotting to confirm the increase or decrease in the gene expression. 

Although a loss-of-function study showed that APL treatment affected various hnRNP family proteins, only hnRNP C was identified as the best target for anticancer activity in human OSCC.

## 5. Conclusions

We observed that APL downregulated the expression of hnRNP family proteins, impairing the ability of hnRNPs to shuttle between the nucleus and cytoplasm and ultimately causing their retention in the cytoplasm. This novel phenomenon has not been previously reported and provide valuable insights into the biological effects of APL. These results warrant further investigation of APL as a source of pharmacologically active agents, identification, and purification of bioactive compounds [2] from its methanol extract and other fractions, and assessment of their antitumor therapeutic efficacy in vivo using experimental preclinical tumor models. 

## Figures and Tables

**Table 1:** Real-time PCR Primer Sequences.

Gene	Forward (5′–3′)	Reverse (3′–5′)
hnRNP A2B1	ATTGAGGCCATTGAATTGCCA	GGCCACCTTGATCTCACACTT
hnRNP H1	GAGGACTTCCCTTTGGATGTAG	ATACCTGTGCCCTATTCTTTCC
hnRNP L	TTCTGCTTATATGGCAATGTGG	GACTGACCAGGCATGATGG
hnRNP K	CCTATGACAGAAGAGGGAGAC	CCCTGTGGTTCATAAGCCATC
hnRNP C	GTACCTCCTCCTCCTCCTATTG	CTGGGTCAGCTCCTTCTTAATG
β-actin	GGCACCCAGCACAATGAAG	TGCGGTGGACGATGGAGG

**Table 2:** si-RNA Sequences.

siRNA	Sequences
hnRNP A2B1	GGAACAUCACCUUAGAGAUTTAUCUCUAAGGUGAUGUUCCTT
hnRNP H1	GCUCAAGGUAUUCGUUUCATTUGAAACGAAUACCUUGAGCTT
hnRNP L	GCAGCCGACAACCAAAUAUTTAUAUUUGGUUGUCGGCUGCTT
hnRNP C	CGUCAGCGUGUAUCAGGAATTUUCCUGAUACACGCUGACGTT
